# Retraction Note: DNA barcoding detects contamination and substitution in North American herbal products

**DOI:** 10.1186/s12916-024-03504-x

**Published:** 2024-07-04

**Authors:** BMC Medicine Editorial Office

**Affiliations:** The Campus 4 Crinan Street, London, N1 9XW UK

**Retraction Note:** **BMC Med 11, 222 (2013)**

**https://doi.org/10.1186/1741-7015-11-222**

The Editor has retracted this article. An investigation by the University of Guelph has found evidence of data fabrication in relation to this article. The Editor therefore no longer has confidence in the presented data.

Steven Newmaster, Dhivya Shanmughanandhan, Subramanyam Ragupathy and Sathishkumar Ramalingam disagree with this retraction. Meghan Grguric has not explicitly stated whether they agree with this retraction.

